# Differences in Mental Health Status Among Asian Americans During the COVID-19 Pandemic: Findings from the Health, Ethnicity, and Pandemic Study

**DOI:** 10.1089/heq.2022.0029

**Published:** 2022-06-24

**Authors:** Biplav Babu Tiwari, Donglan “Stacy” Zhang

**Affiliations:** ^1^ Department of Health Policy and Management, College of Public Health, University of Georgia, Athens, Georgia, USA.; ^2^ Division of Health Services Research, New York University Long Island School of Medicine, Mineola, New York, USA.

**Keywords:** Asian Americans, COVID-19, mental health

## Abstract

**Purpose:**

This study aims to explore the differences in mental health status among Asian Americans during the COVID-19 pandemic.

**Methods:**

Data from the 2020 Health, Ethnicity, and Pandemic (HEAP) Study were used to explore the psychological distress of 2672 adults, using the Standard Kessler Psychological Distress Scale.

**Results:**

We observed that among Asian American subgroups, South Asian Americans had significantly higher odds of experiencing psychological distress than non-Hispanic White Americans (odds ratio = 1.82, 95% confidence interval = 1.00–3.31), after controlling for covariates.

**Conclusion:**

The study identified differences in mental health status among Asian American subgroups. We recommend the implementation of culturally appropriate interventions to help Asian Americans cope with mental health challenges.

## Introduction

Mental health disorders were a significant burden worldwide even before the COVID-19 pandemic, where depression ranked second and anxiety eighth for all causes of disability globally in 2019.^1^ The situation has been further exacerbated by the trauma and stress stemming from the pandemic as well as the quarantines and social distancing practices.^2^ A survey conducted by the Centers for Disease Control and Prevention (CDC) found that the prevalence of symptoms of anxiety disorder increased by three times and that of depressive disorder by four times in April to June 2020 than that reported in the second quarter of 2019.^3^

Though all the people are possibly affected by the virus, a plethora of emerging evidence suggests that the pandemic has a disproportionately larger impact on ethnic minorities, including Asian Americans.^2,4–7^ A survey study conducted using the four-item Patient Health Questionnaire found that Hispanics and African Americans had a higher prevalence of anxiety or depressive disorder compared with the non-Hispanic whites during the COVID-19 pandemic.^3^ Similarly, the CDC reported that the symptoms of current depression were 59% more frequently in Hispanics than in their non-Hispanic white counterparts.^8^

Moreover, there has been a sevenfold increase in depression and anxiety prevalence among Asian Americans during the COVID-19 pandemic compared with 2019.^7^ These differences in mental health impacts can be attributable to the combined effects of an increase in racial discrimination, social isolation, and already existing underutilization of mental health care among these vulnerable groups across the United States.^4,6,7,9^

However, Asian Americans are an extremely heterogeneous group, and it is unknown which subgroups within Asian Americans are mostly affected. Before the pandemic, evidence shows a huge variation in mental health impact among Asian American subgroups. For example, a study based on data from New York City found that Southeast Asians had the greatest depression risk compared with other Asian American subgroups.^10^ A meta-analysis found that the prevalence of depression was the highest among Filipino Americans compared with other racial and ethnic groups.^11^ These data suggested that the mental health status among Asian American subgroups differed substantially, and tailored interventions were needed to meet the specific demands of the subgroups to mitigate the research and intervention gaps. Thus, this study explores the difference in mental health status within the Asian American subgroups during the COVID-19 pandemic using data from a nationally representative survey.

## Materials and Methods

Data from the 2020 Health, Ethnicity, and Pandemic (HEAP) Study were used. The study was conducted by the National Opinion Research Center (NORC) at the University of Chicago in October 2020, and the study sample was drawn from AmeriSpeak Panel using sampling strata, including age, gender, race/ethnicity, and educational attainment, following the American Association for Public Opinion Research guidelines and additional minority respondents from Dynata's nonprobability online opt-in panel. The full sample included 2709 adults aged ≥18 years in which Asians were oversampled (*n*=979) to investigate how minorities, especially Asians, were affected by the pandemic.

The Asian American group was divided into three subcategories: East Asians (Chinese, Japanese, and Korean), Southeast Asians (Burmese, Cambodian, Filipino, Hmong or Miao, Indonesian, and Vietnamese), and South Asians (Indian, Bangladeshi, Nepalese, and Pakistani). The study was approved by the Institutional Review Board at the NORC.

The outcome measure was psychological distress, which was assessed with the Kessler Psychological Distress Scale (K6), a validated and widely used self-report measure of moderate psychological distress. The K6 measures psychological distress with six questions (e.g., “How often did you feel nervous?” “How often did you feel worthless?”) scored on a five-point Likert scale ranging from none of the time to all of the time.^12^ It ranged from 0 to 24, with a high value indicating a high level of psychological distress.^12^

According to Kessler et al., the optimal cutoff point on the K6 was 0 to 12 versus 13 or more, because at this cutoff point, the total classification accuracy for severe psychological distress could be reached at 0.92 (0.02).^13^ Thus, a score of 13 and above indicated serious mental distress in the present study.^12,14^ Data regarding sociodemographic factors and health behaviors were also collected, including sex, age; race; marital status; educational attainment; annual household income; employment status; insurance status; self-rated health status; and residential region.

Observations with missing values were first assessed and dropped from the analytic sample. Descriptive analysis was performed for the sociodemographic characteristics of the population and regression analysis was performed to explore the association between racial/ethnic groups and psychological distress, adjusting for the aforementioned covariates. All estimates were weighted to account for the complex sampling design and to provide unbiased estimates for the U.S. adult population.

## Results

Overall, there were 37 (1.37%) observations with missing values: race (*n*=6, 0.22%), insurance (*n*=29, 1.07%), and health status (*n*=2, 0.07%). We dropped the observations with missing values and used the complete sample for analysis. The characteristics of the study sample are shown in Table 1. The sample of 2672 participants were predominately young and middle-aged (46.02% in the age group of 18–44 years), female (51.69%), employed (58.58%), married (58.09%), and living in the South (37.85%). The majority of the participants had at least some college education (34.23%) and earned between $50,000 and $99,999 (34.52%). Similarly, more than half of the participants were enrolled in private insurance (54.44%) and had self-reported excellent or good health status (54.46%).

**Table 1.** Characteristics of the Study Sample (*N*=2672)

	Total	Non-Hispanic whites	Non-Hispanic blacks	Hispanics	East Asians^a^	Southeast Asians^b^	South Asians^c^	Other races	***p***
	***N* =2672**	***N* =514**	***N* =590**	***N* =532**	***N* =517**	***N* =218**	***N* =183**	***N* =149**	
Psychological distress (%)	16.68	14.99	16.82	22.59	12.30	28.61	23.09	15.34	0.0247
Age group (%)									<0.001
18–44	46.02	40.05	53.00	58.81	44.87	62.33	67.90	53.00	
45–64	32.53	33.52	30.07	29.73	33.97	24.27	23.37	40.29	
65+	21.45	26.43	16.93	11.46	21.16	13.40	8.73	6.72	
Female (%)	51.69	51.20	54.47	49.84	54.26	57.63	43.37	56.72	0.5736
Educational attainment (%)									<0.001
Less than high school	9.70	11.15	24.45	7.52	10.93	6.65	2.06	6.16	
High school	28.31	32.77	32.75	14.68	28.48	7.40	20.59	27.91	
Some college	27.76	30.41	24.76	20.63	17.11	13.92	24.67	29.15	
College and above	34.23	25.67	18.05	57.17	43.48	72.04	52.67	36.78	
Household income (%)									<0.001
Less than $24,999	18.87	13.75	35.36	28.44	11.67	21.90	15.69	15.75	
$25,000 to $49,999	22.52	21.68	25.04	26.21	22.21	17.54	12.06	18.36	
$50,000 to $99,999	34.52	36.84	28.48	31.54	31.03	38.88	29.38	31.58	
$100,000 or more	24.09	27.73	11.12	13.81	35.09	21.67	42.87	34.31	
Employed (%)	58.58	58.58	58.58	58.58	58.58	58.58	58.58	58.58	0.3908
Married (%)	58.09	63.62	37.71	54.49	57.48	45.77	62.77	51.56	<0.001
Insurance status (%)									<0.001
Private insurance	54.44	56.28	47.32	47.16	60.21	50.87	60.58	70.98	
Medicare	22.71	25.26	18.75	19.72	25.72	21.35	12.43	9.33	
Medicaid	14.11	10.65	25.38	19.27	10.29	15.99	18.05	14.20	
Uninsured	8.73	7.81	8.55	13.85	3.78	11.79	8.93	5.49	
Self-rated health status (%)									0.0014
Excellent or good health	54.46	56.97	42.41	49.08	64.41	60.99	64.72	59.78	
Good health	31.56	29.88	38.69	37.78	23.45	26.18	24.19	21.87	
Fair or poor health	13.98	13.15	18.90	13.14	12.14	12.82	11.09	18.35	
Residential region (%)									<0.001
Northeast	17.33	18.56	15.38	14.52	21.30	12.38	30.58	10.69	
Midwest	20.82	26.21	16.58	8.74	11.18	11.82	15.29	12.81	
South	37.85	34.39	58.73	37.35	16.14	27.09	35.02	53.05	
West	24.00	20.84	9.31	39.39	51.38	48.71	19.12	23.46	

^a^ East Asians include Chinese, Japanese, and Korean.

^b^ Southeast Asians include Burmese, Cambodian, Filipino, Hmong or Miao, Indonesian, and Vietnamese.

^c^ South Asians include Indian, Bangladeshi, Nepalese, and Pakistani.

The sociodemographic characteristics of different racial/ethnic groups were similar. Among all the racial/ethnic groups, the majority of the population were aged 18–44 years, female except for South Asian respondents (43.37%) and Hispanic respondents (49.84%), with at least some college education except for non-Hispanic Black (32.07% with high school education) and non-Hispanic whites (32.77% with high school education), employed, married except for non-Hispanic Black (37.71% married) and Southeast Asian respondents (45.77%), enrolled in private health insurance, had self-reported excellent or good health, and living in the South except for Hispanics, East Asian, and Southeast Asian respondents (39.39%, 51.38%, and 48.71% living in the West respectively).

However, in terms of household income, the majority of the East and South Asian Americans had a household income of $100,000 or more, that is, 35.09% and 42.87%, respectively, non-Hispanic Black respondents had a household income of less than $25,000 (35.36%), and the rest had between $50,000 and $99,999. Importantly, however, there were no systematic differences with regard to sex and employment status.

**FIG. 1.**
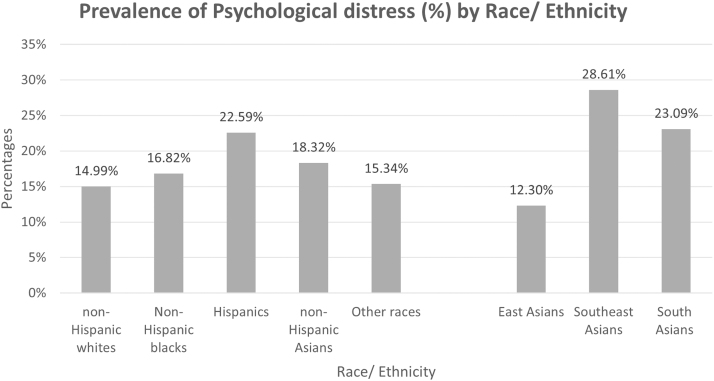
Prevalence of psychological distress by race/ethnicity.

Figure 1 displays the prevalence of psychological distress among the different racial/ethnic groups. We observed that the Hispanic population had the highest prevalence of psychological distress (22.59%), followed by non-Hispanic Asian Americans (18.32%). Within the non-Hispanic Asian subgroups, a higher prevalence of psychological distress among the Southeast Asian (28.61%) and South Asian Americans was observed (23.09%), respectively.

The unadjusted regression analyses demonstrated significant associations between being Hispanic (unadjusted odds ratio [OR]=1.66, 95% confidence interval [CI]=1.10–2.49, *p*=0.015), South Asian (unadjusted OR=1.70, 95% CI=1.03–2.82, *p*<0.001), or Southeast Asian (unadjusted OR=2.27, 95% CI=1.38–3.73, *p*=0.039) and psychological distress, whereas the adjusted analysis revealed that compared with non-Hispanic White respondents, South Asian Americans was significantly associated with psychological distress (adjusted OR=1.82, 95% CI=1.00–3.31; *p*=0.05) after controlling for covariates (Table 2).

**Table 2.** Regression Analysis Between Race and Mental Health

	Psychological distress							
	Unadjusted				Adjusted^a^			
	OR	95% CI		***p***	OR	95% CI		***p***
Race groups (ref. non-Hispanic whites)								
Non-Hispanic blacks	1.15	0.76	1.73	0.511	0.68	0.42	1.09	0.108
Hispanics	1.66	1.10	2.49	0.015	1.08	0.65	1.78	0.767
East Asians^b^	0.80	0.52	1.21	0.285	0.96	0.59	1.58	0.877
Southeast Asians^c^	2.27	1.38	3.73	0.001	1.78	0.99	3.19	0.053
South Asians^d^	1.70	1.03	2.82	0.039	1.82	1.00	3.31	0.05
Other races	1.03	0.45	2.32	0.948	0.90	0.42	1.96	0.796

^a^ The model adjusted for age, gender, educational attainment, household income and size, citizenship, employment status, marital status, region, insurance status, and self-rated health, and sampling weights.

^b^ East Asians include Chinese, Japanese, and Korean.

^c^ Southeast Asians include Burmese, Cambodian, Filipino, Hmong or Miao, Indonesian, and Vietnamese.

^d^ South Asians include Indian, Bangladeshi, Nepalese, and Pakistani.

CI, confidence interval; OR, odds ratio.

Thus, within the Asian American subgroups, we observed that South Asians and to some extent Southeast Asians (adjusted OR=1.78, 95% CI=0.99–3.19; *p*=0.053) were more likely to experience psychological distress during the COVID-19 pandemic, compared with the non-Hispanic White population.

## Discussion and Conclusion

In this study, we assessed the difference in the prevalence of psychological distress among the various racial/ethnic groups during the COVID-19 pandemic in a nationally representative sample of the United States. Unadjusted regression analysis demonstrated that Hispanic, South Asian, and Southeast Asian populations had higher odds of experiencing psychological distress than the non-Hispanic white population.

The COVID-19 pandemic disproportionately affects the mental health of racial/ethnic minorities.^6^ In line with our study, much evidence has been generated that shows the worse mental health among the Hispanic and African American populations during the COVID-19 pandemic.^3,8^ The social determinants of health such as low income, having less than $5000 in savings, unemployment, no health insurance, and language and cultural barriers were found to be associated with poor mental health among Hispanic and African American groups during the COVID-19 pandemic.^8,15–17^

However, less priority has been provided to study mental health among Asian Americans and especially among Asian subgroups amid the COVID-19 pandemic.^18^ Even before the pandemic, Asian Americans had poor mental health status.^10,11,18–20^ The current climate of racial discrimination and social isolation has further worsened the condition.^2,4,6^ Our result of South Asians and Southeast Asians being more likely to experience psychological distress during the COVID-19 pandemic was consistent with the study by Woo and Jun,^7^ which found a sevenfold increase in the prevalence of depression and anxiety symptoms among Asian Americans.

Similarly, a study conducted using the patient health questionnaire eight-item depression scale (PHQ-8) found that, during the pandemic, the prevalence of depression among the South Asians was three times higher than among the Chinese Americans (38% vs. 13%).^21^ In addition, another study found that Vietnamese respondents were most vulnerable to mental distress.^22^ These increases in mental health impacts can be attributed to stress due to migration and acculturation, somatization, stigmas surrounding mental health and use of mental health services, underrepresentation among the mental health care providers, lack of culturally informed interventions and social isolation, and increased racial discrimination amid the COVID-19 pandemic.^2,18–20^

The study has several imitations. Due to the self-reported responses from the participants, the study was subject to recall bias and measurement errors. Also, we could not establish a causal relationship as data were obtained from a cross-sectional survey. Finally, we were not able to rule out the potential selection bias given that only English and Spanish languages were used in the survey and Asian languages were not used. It limited the inclusion of minority groups who may have language barriers or different cultural backgrounds that may lead to underreporting of mental health challenges, compromising the generalizability of the study.

In addition, self-rated health status might be a mediator linking race/ethnicity to mental health, but mental health status could also influence overall health status. We did not conduct a formal mediation analysis, but future studies are warranted when longitudinal data become available. Despite these limitations, the study is unique in terms of its aim to assess the disparities in mental health among the Asian American subgroups during the COVID 19 pandemic and would inform future research to develop effective interventions.

In conclusion, in a nationally representative sample of the United States, race/ethnicity was strongly associated with psychological distress. Southeast Asians and South Asians may be most affected in the present context of the pandemic and need the attention of the policymakers to develop effective and culturally appropriate interventions based on data-driven evidence to uplift their condition.

## Implications for Policy and Practice

The COVID-19 pandemic resulted in a surge of mental health challenges, particularly among minorities. Asian Americans face acculturation stress and discrimination that is associated with psychopathology, which ultimately results in low utilization of mental health services.^23^ Thus, the study suggests that policy makers should develop comprehensive, effective, and culturally appropriate interventions to help Asian American subgroups cope with mental health challenges. Moreover, our research and evidence suggest a disproportionate impact on mental health among Southeast Asians and South Asians, so these interventions need to be tailored to ensure health equity.

Further, during the study, we found only a handful of the evidence that evaluates the impact of mental health among Asian Americans and its subgroups. So, studies on Asian Americans and their subgroups might help to generate better evidence on their real-life condition and guide the program interventions accordingly to improve their quality of life.

## Abbreviations Used

CDC: Centers for Disease Control and Prevention
CI: confidence interval
K6: Kessler Psychological Distress Scale
NORC: National Opinion Research Center
OR: odds ratio
PHQ-8: patient health questionnaire eight-item depression scale
